# The alleviative effects comparison of four flavonoids from bamboo leaves on ulcerative colitis in an Alzheimer mouse model

**DOI:** 10.1111/cns.14620

**Published:** 2024-02-09

**Authors:** Taiyu Zhang, Cuicui Jia, Longyi Ran, Jiarui Shi, Tsendsuren Amarmend, Huiying Li

**Affiliations:** ^1^ College of Biological Sciences and Technology, Beijing Key Laboratory of Food Processing and Safety in Forestry Beijing Forestry University Beijing China

**Keywords:** Alzheimer's disease, bamboo leaf flavonoids, homoorientin, ulcerative colitis

## Abstract

**Background:**

Clinically, patients with dementia are at high risk of developing enteritis, especially those with AD. This study explored the potential therapeutic benefits of bamboo leaf flavonoids (BLF) for ulcerative colitis (UC) treatment in Alzheimer's disease (AD) mouse model.

**Methods:**

Various methods were employed, including pathological staining of brain/colon tissue, inflammatory cytokine detection in serum, and oxidative stress indicator assessment to compare ulcerative enteritis (UC) injury in normal and AD mice and determine whether AD mice were susceptible to colitis. Then, the effects of BLF on UC and AD were investigated via several unique indices further to determine whether it alleviated colitis injury and possessed beneficial properties. Moreover, four main components of BLF were utilized to treat primary colon epithelial cells and neuron cells to compare their effects in alleviating inflammation and oxidation. Furthermore, homoorientin embedded with ursolic acid was detected by HPLC and the in vitro release simulation experiments of the nanoparticles were performed.

**Results:**

BLF complexes positively impacted ulcerative colitis by reducing disease activity, it also helped to reduce inflammation. Moreover, the BLF complexes decreased oxidative stress in the brain and colon tissues, indicating its potential as a neuroprotective agent. The flavonoid complexes reduced the expression levels of GFAP, Iba‐1, and Aβ in the brain tissue, highlighting its role in attenuating neuroinflammation and AD pathology. Additionally, the embedded homoorientin coated with ursolic acid showed stronger bioactivities when compared with the uncoated group.

**Conclusion:**

These results suggest that BLF complexes and its four main chemicals may be useful for treating AD‐ and UC‐related complications, the embedded homoorientin coated with ursolic acid even demonstrated stronger bioavailability than homoorientin. Considering BLF complexes were verified to suppress the progressions of AD and UC for the first time, and the embedded homoorientin was never reported in published articles, the present study might provide a new perspective on its potential applications.

## Graphical abstract

Alzheimer's disease (AD) mice are more susceptible to ulcerative colitis (UC). BLF and four main chemicals are useful for treating AD and UC, especially homoorientin. Especially, the coated homoorientin nanoparticles demonstrated a stronger bioavailability in present model.

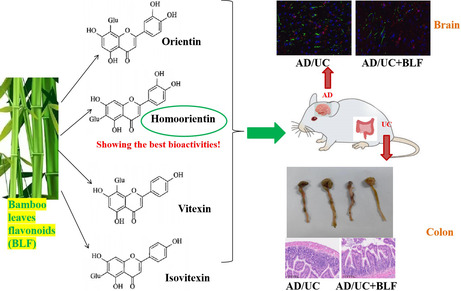

## 1 INTRODUCTION

Bamboo leaves contain many active ingredients with medicinal benefits and more than 100 chemical compounds, including flavonoids and flavonoid glycosides, volatile components, phenolic acids, and polysaccharides.^1^ Bamboo leaf flavonoids (BLF) have attracted considerable recent attention, with orientin (OT), homoorientin, vitexin (VT), and isovitexin (IsVX) representing the four primary forms.^2^, ^3^ Flavonoids can alleviate neuronal degeneration since they demonstrate activity in the central nervous system and successfully cross the blood–brain barrier, like Alzheimer's disease (AD).^4^ In particular, recent studies have shown that bamboo leaf extract and its constituents display significant potential for preventing infectious, inflammatory, oxidation, cardiovascular, metabolic, and neurological neuropsychiatric diseases.^5^, ^6^, ^7^ Increasing evidence shows that consuming flavonoid‐rich food benefits normal cognitive function.^3^, ^8^ Studies have shown that individuals who routinely consume more flavonoids daily are less likely to develop neurodegenerative diseases.

AD is a neurodegenerative disease with insidious onset and progressive impairment of behavioral and cognitive functions, including memory, comprehension, language, attention, reasoning, and judgment, typically affecting older people.^9^ Since no treatment exists that can delay AD progression,^10^ this disease severely threatens human health and quality of life and imposes a significant burden on families and society. Clinically, patients with dementia are at high risk of developing enteritis, especially those with AD. The gut–brain axis (GBA) is a nerve–endocrine‐mediated bidirectional response system between brain and gut, which acts on the communication between gut microbes and brain.^11^, ^12^ Related studies revealed that when the intestinal flora was abnormally altered, the pathogenic bacteria, inflammatory factors, and oxidative stress responses produced by its metabolites increased the permeability of the intestinal epithelial barrier and the blood–brain barrier, causing a chronic inflammatory response in the brain.^13^, ^14^ Several basic and clinical studies confirmed a close relationship between intestinal flora disorder and AD, while the intestinal microflora changes affected the function of the “microbe‐GBA,” further impacting AD progression.^15^, ^16^, ^17^ Some natural products, such as flavonoids, have shown a beneficial alleviative effect on disease progression.^18^ Although extensive research has examined the correlation between flavonoids and AD, minimal studies are available regarding BLF. It is vital for future studies to thoroughly explore the development of medicinal drugs from BLFs. Furthermore, it is crucial to investigate the intricate mechanisms underlying these drugs to potentially provide effective treatment for those affected by AD.

## 2 MATERIALS AND METHODS

### 2.1 Materials and reagents

The SPF male mice were purchased from the Huachuangxinnuo Company (Nanjing, Jiangsu province, China) with the permission number (SCXK 2020–0009). The BLF (purity ≥ 98%) and the four main chemicals were obtained from J&K Scientific (Beijing, China) and Solarbio (Beijing, China). Four types of inflammatory cytokine (TNF‐α, IL‐1β, IL‐6, and IFN‐γ) enzyme‐linked immunosorbent assay (ELISA) kits were purchased from Saipei Technology Company (Wuhan, Hubei province, China), while the malondialdehyde (MDA), glutathione peroxidase 4 (GPX4), and 4‐hydroxynonenal (4‐HNE) detection kits were supplied by Beyotime Biotechnology (Nanjing, Jiangsu province, China). The colon tissue hematoxylin–eosin (HE) staining kit and brain tissue‐specific staining kits (GFAP/Iba‐1 fluorescence double staining, Aβ, and Nissl corpuscle) were acquired from Solarbio (Beijing, China).

### 2.2 AD mouse feeding and UC model construction

Based on our previous toxicokinetic experiment results, we found the BLF dosage of 1 mg/kg body weight (b.w.) showed weaker bioactivities in alleviating UC injury when compared with the one of 2 mg/kg b.w. group, and the BLF of 3 mg/kg b.w. seemed to cause tachycardia in several mice, even worse, the BLF of 5 mg/kg b.w. demonstrated the obvious side effects, like liver toxicity. Thus, considering both efficacy and toxic effects, the proper dosage of BLF in mice model was selected as 2 mg/kg b.w. The first part of animal experiments was performed as follows: 20 male APP/PS1 transgenic mice (3 months old) were randomly divided into four groups (five mice per group): APP control without any treatment, UC model group, BLF treatment group (2 mg/kg b.w.), and UC mice flavonoid treatment group, *n* = 5. 20 male B6C3 control mice (3 months old) were randomly divided into four groups (five mice per group): normal control without any treatment, UC model group, BLF treatment group (2 mg/kg b.w.), UC mice flavonoid treatment group, *n* = 5. The mice in the UC model group received dextran sulfate sodium salt (DSS) solution (3%, in ddH_2_O) for 3 weeks, while those in the BLF treatment group were orally gavaged daily for 4 weeks.

The second part of animal experiments was performed as follows: 30 male APP/PS1 transgenic mice (3 months old) were randomly divided into six groups (five mice per group): APP control without any treatment, UC model group, homoorientin treatment group (2 mg/kg b.w.), embedded homoorientin group (2 mg/kg b.w.), UC mice homoorientin treatment group, and UC mice embedded homoorientin treatment group, *n* = 5. The mice in the UC model group received DSS solution (3%, in ddH_2_O) for 3 weeks, while those in the homoorientin treatment group were orally gavaged daily for 4 weeks.

All the mice were sacrificed at 29 days, and the blood samples and the organs were collected for the further detections.

### 2.3 The detection of the organ index, hematological parameters of the mouse blood, disease activity indices of the UC mice, and colon length

The organs (hearts, livers, kidneys, lungs, spleens, thymuses, and intestines) were separated and weighed, *n* = 5. The colon tissue lengths were measured and compared according to the reference.^19^ Small pieces of liver, kidney, heart, and intestinal tissue were immersed in 10% formalin for 48 h, embedded in paraffin, and cut into slices using a slicer (Leica, Germany). The slices were subjected to HE staining and observed under an optical microscope (Olympus, Japan). Blood samples were collected from the posterior orbital plexus, half of which was placed in a test tube containing heparin for hematological detection (whole blood). The colitis disease activity indices (DAI) was calculated using (weight loss + stool traits + bleeding)/3, *n* = 5 (Table S1).

### 2.4 Detection of inflammatory cytokines in mice serum, brain, and colon tissues

The IL‐1β, IL‐6, TNF‐α, and INF‐γ concentrations in mice serum, brain, and colon tissues were quantified using an ELISA kit according to the instructions of the manufacturer (*n* = 5). A pore plate was utilized in a multifunctional enzyme label instrument, such as a microplate reader, to quantify the results by measuring the light absorbance of each pore in the plate at 450 nm. The subsequent optical density value reflected the number of cytokines bound to the membrane and could be used to determine their concentrations in the mice serum and tissues samples.

### 2.5 Detection of the MDA, GPX4, and 4‐HNE in the brain and colon tissue homogenates

The brain and colon tissues were subjected to routine homogenate treatment and centrifuged at 4°C and 10,000 g for 10 min, after which the supernatant was placed on ice for testing. The supernatant was incubated with thiobarbituric acid at 95°C for 60 min and cooled on ice for 10 min. The absorbance of the supernatant was observed at the wavelength of 532 nm. The GPX4 and 4‐HNE in the brain and colon tissue homogenates were detected according to the protocols, using specific ELISA kits (*n* = 5).

### 2.6 Pathological staining of the brain and colon tissues

During HE staining, slices of the mouse colonic tissue were bounded in 4% paraformaldehyde, dehydrated, embedded in paraffin, cut into pieces, stained, and sealed with neutral gum. The morphology of the colonic mucosa was examined using an optical microscope (*n* = 5).

### 2.7 Specific marker staining of the brain tissue (GFAP/Iba‐1 fluorescence double staining, Aβ, Nissl corpuscle)

During GFAP/Iba‐1 fluorescence double staining, the tissue was blocked with 5% NGS/2% BSA/1.5% Triton/PBS, followed by overnight incubation with the anti‐Iba‐1 or anti‐GFAP antibody at 4°C. The tissue sections were processed concomitantly at consistent microscope settings to clarify the intensity variability based on staining or imaging differences, while the individual markers of all the tissues were immunolabeled to reduce run‐to‐run variability. Visualization was performed using a Nikon Eclipse 800 microscope (Nikon, Tokyo, Japan) equipped with an Olympus DP71 camera (Olympus, Center Valley, PA, USA).

Nanogold (AuNPs) was used as a colorimetric probe, while the aptamer (aptamer@AuNPs) adsorbed by the AuNPs denoted the Aβ 40 binding element. Aptamer@AuNPs accumulation appeared blue‐purple in high salt conditions. Aβ was added, which combined with the aptamer to create a complex attached to the AuNPs surface, increasing the salt tolerance of the nanoparticles to maintain AuNPs dispersion. The solution turned pink, allowing quantitative detection by comparing the absorption intensity ratio of the solution before and after Aβ addition at a limit of detection (LOD) of 10 nmol/L.

The specific Nissl staining procedure started with conventional dewaxing to water (xylene I and xylene II, respectively, for 15 min, followed by gradient alcohol dehydration with 100% I, 100% II, 95%, 90%, 80%, 70%, and 50%, respectively, for 5 min). Each sample was flushed three times in distilled water for 5 min each, after which they were placed in a warm box at 60°C with 1% toluidine blue for 40 min (or with tar violet for 30 s). After removing the dye by washing with distilled water, the samples were dehydrated in 70%, 80%, 95%, and 100% ethanol, rendered transparent using xylene, and sealed with neutral gum.

### 2.8 Isolation of the primary neuronal and colon epithelial cells of the AD mice with UC

The anesthetized AD mice with UC were transcardially perfused with cold PBS. Hippocampal tissue was isolated from the mouse brains, placed in 2 mL of ice‐cold Hank's Balanced Salt Solution (HBSS), and mechanically dissociated using a scalpel to be as small as possible. Only DMEM medium was added to the tube. The tissue clumps were converted into a cell suspension via low‐speed centrifugation (500 rpm for 5 min), washed with 2 mL HBSS, and mechanically homogenized using a scraper. The cell suspension was passed through a 70 μm cell strainer to remove debris. The filters were washed four times with 1 mL HBSS to ensure adequate cell collection via low‐speed centrifugation (500 rpm for 5 min). The cells were then cultured in a DMEM medium with 10% FBS.

Colon tissue was removed from the anesthetized AD mice with UC and minced. After repeated cleaning, the supernatant was discarded. Next, 10 mL type I collagenase (0.1%) and hyaluronidase (0.1%) were added to the tube for digestion at 37°C for 25 min, after which the epithelial cells were isolated. Then the supernatant was removed, and the precipitate was added to the growth medium. After three centrifugation cycles (1250 rpm for 5 min), the cell precipitates were seeded into the DMEM medium containing 10% FBS, which was changed every 24 h. When the cell density reached about 70%, the next BLF treatment was initiated.

### 2.9 The effect of the main BLF components on the inflammatory cytokines and oxidative markers in the primary colon epithelial and neuronal cells of the AD mice with UC

To investigate the potential of the key BLF components as a therapeutic approach for AD mice with UC, this study utilized four main chemicals (OT, homoorientin, VT, and IsVX) to treat the primary colon epithelial and neuronal cells, respectively. The appropriate dosages of the four chemicals were selected, after which the inflammatory cytokines and oxidative markers in the two cell types were measured, aiming to compare the most effective BLF components (*n* = 5).

The two types of cells were treated at four chemicals for 48 h, respectively, and their dosages were between 0 and 500 μm, after which a CCK‐8 kit was used for cell viability detection (*n* = 5). Based on the main principles of dosage selection, the cell viability ≥80%, with statistical differences between the control and treatment groups. Next, the inflammatory cytokines and MDA, GPX4, and 4‐HNE oxidative markers in the two types of cells were determined using ELISA kits (*n* = 5).

### 2.10 Detection of embedded homoorientin by HPLC and invitro release simulation experiments of the nanoparticles

Embedding methods: 10 mg/mL ursolic acid solution was prepared, 2 mg homoorientin was weighed and dissolved in 1 mL ursolic acid methanol solution. 1 mL ursolic acid and homoorientin mixed solution was added drop by drop under vortex condition, then 2.5 mL PVA solution with concentration of 2.5% was added into the tube and mixed evenly. After ultrasonic emulsifying for 90s, the mixture was quickly transferred into 35 mL PVA solution with the concentration of 0.3%, which was continuously stirred on magnetic stirrer for 12 h later. Next, the sample was collected in a centrifuge tube at 12,000 r/min and centrifuged at 4°C for 30 min, and the precipitate was the homoorientin embedded in nanoparticles.

To verify the embedding effect, methanol was used to break down the nanoparticle structure, and the presence of flavonoids was detected by high‐performance liquid chromatography (HPLC). The Agilent 1290 liquid chromatograph was used to detect homoorientin under the following liquid phase conditions: Mobile phase: 0.2% formic acid water (A) – acetonitrile (B), with a flow rate of 0.2 mL/min. Gradient elution conditions: 0–0.5 min, 30% B; 0.5–2 min, 30%–80% B; 2–3 min, 80%–98% B; 3–5 min, 98% B; 5–7 min, 98%–30% B; 7–9 min, 30% B; 9 min, stop. Injection volume: 2 μL. Column temperature: 35°C, detection wavelength: 330 nm. The obtained precipitate was dissolved in 3 mL of methanol (chromatographic grade) to obtain the sample.

Further to investigate the release performance of nanoparticles, we then investigated the release of nanoparticles in simulated gastric juice and intestinal fluid. In gastric juice detection course, after suspending the freshly prepared nanoparticles with appropriate amount of deionized water, simulated gastric juice (0.2 g NaCl, 1 g Pepsin, 0.7 mL concentrated hydrochloric acid, pH = 1.2) was added into the nanoparticles solution with a ratio of 2:1. The mixture was incubated in a water bath at 37°C and stirred with magnetic force at a frequency of 100 r/min. Similarly, in intestinal fluid detection course, freshly prepared nanoparticles were suspended in an appropriate amount of deionized water and mixed with simulated intestinal fluid (0.6 g KH_2_PO4, 0.2 M NaOH, 1 g trypsin, 0.5 g pig bile salt, pH = 7.0) in a 1:2 ratio. The mixture was incubated in a water bath at 37°C and stirred with magnetic force at a frequency of 100 r/min. Every 30 min,1 mL of the mixture was taken and centrifuged at 4°C at 12,000 r/min for 30 min, then it was dissolved in methanol solution, and we detected the content of homoorientin by HPLC.

### 2.11 Statistical analysis

All the data were presented as mean ± standard deviation (SD), which were analyzed using the SPSS 18.0 and GraphPad Prism 6.0 software. The statistical analyses between two groups (control group vs. treatment groups, single treatment group vs. two treatments groups) were performed using Student's *t*‐test. *p* < 0.05 was considered to be statistically significant.

## 3 RESULTS

### 3.1 The organ indexes and hematological parameters in the mouse blood

**FIGURE 1:**
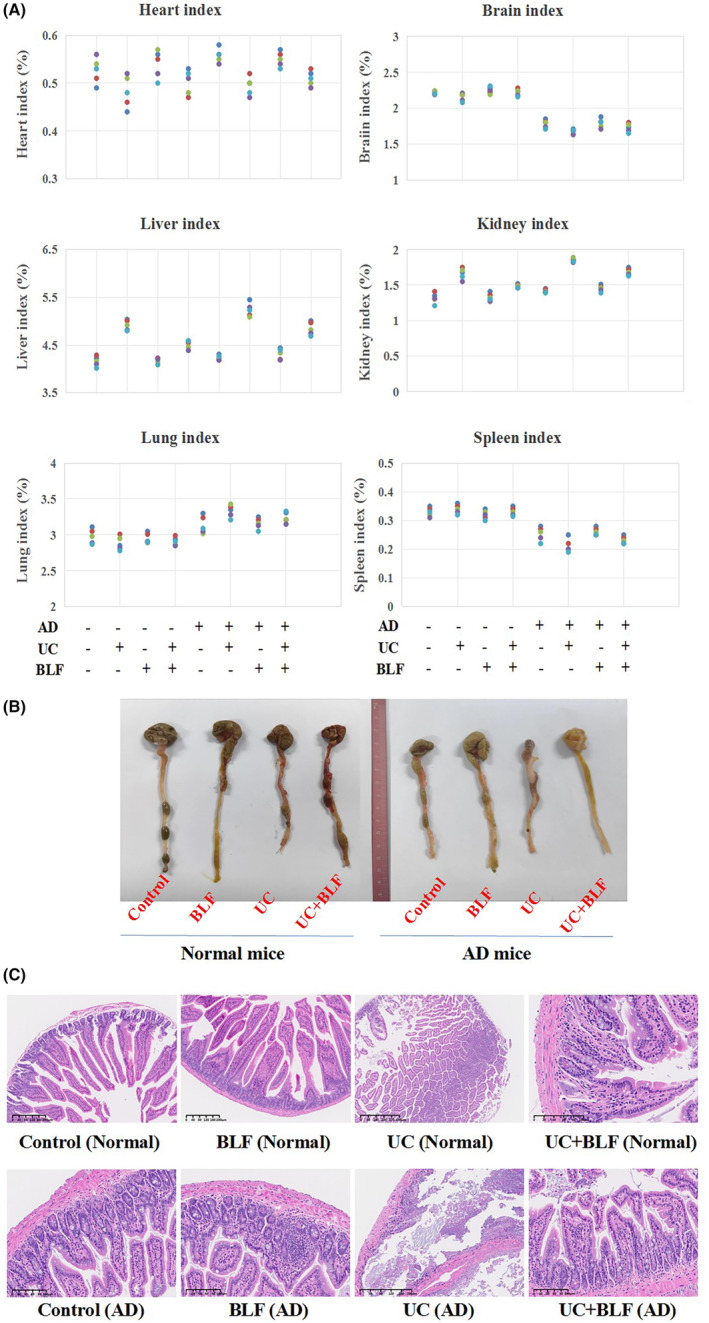
Organ indexes and pathological conditions of the mouse colon tissue. (A) The organ indexes (heart, liver, kidney, spleen, lung, and brain indexes) of the mice in the different groups (%), *n* = 5. (B) The colon tissue lengths and pathological condition of the mice, *n* = 5. (C) HE staining of the colon tissue (200×). UC refers to mice with DSS‐induced colitis, and BLF denotes mice treated with BLF, *n* = 5. *p < 0.05 when compared with the control level and #*p <* 0.05 when compared to the UC level.

The organs (hearts, livers, lungs, spleens, kidneys, and brains) of the animals were weighed (*n* = 5). The results indicated no differences between the lungs of the normal and AD mice, whether exposed to BLF treatment or not. As a digestive and toxic target organ, the liver index in the UC model, both in the normal and AD mice, increased compared with the control group (*p* < 0.05), indicating a possible toxic effect by DSS‐induced UC. Although the liver index decreased slightly compared with the UC group after BLF treatment, it remained higher than the control group (*p <* 0.05), suggesting that BLF might alleviate colitic injury. The kidney index showed a similar tendency in the UC model and BLF groups. However, the brain index in the AD mice with UC decreased compared with the control, which was significantly upregulated by BLF. This verified that BLF might alleviate UC damage to the brain tissue structure to some degree (Figure 1A).

Determination of the hematological parameters in the mouse blood indicated a dramatic increase in the calculated WBC, lymph cell, mid cell, and gran cell values in the UC mice compared with the control (*p <* 0.05), regardless of whether they were in the normal or AD groups (*n* = 5). BLF neutralized these values to some degree (*p <* 0.05), indicating that BLF might alleviate colitic injury by suppressing inflammatory reactions and enhancing immune ability. Furthermore, the RBC and PLT values of the UC model group were lower than the control (*p <* 0.05). BLF significantly upregulated these values (*p <* 0.05), further verifying that BLF could recover colitic injury by strengthening the immune system (Table S2).

### 3.2 The DAI of the mice with UC and colon length determination

The DAI value of the normal mice in the UC group (7.92 ± 0.85) was higher than the control level (0.00 ± 0.13), while that of the UC + BLF group (5.38 ± 0.93) was significantly below the UC level. The AD mice displayed a similar tendency, with the DAI value in the UC group (9.55 ± 1.30) exceeding the control level (0.00 ± 0.25), while that of the UC + BLF group (6.92 ± 1.16) was significantly lower than the UC level (*p <* 0.05). Furthermore, both in normal group and AD group, the colon tissue of UC mice seemed to be inelastic, and the calculated colon tissue lengths of the UC group were shorter than the ones in control and BLF‐treated groups, while that of the UC + BLF group was longer than the one in UC group, regardless of whether in normal mice or AD mice (Figure 1B, *n* = 5).

### 3.3 Pathological colon tissue results

Referring to successful UC model construction, we found that UC mice were always in diarrhea conditions, their average bodyweight was the lowest one among these groups, and mice colon tissue in UC group was obviously different with the control, the intestinal wall was thicker and lacked elasticity. Meanwhile, HE staining indicated edema, occasional hemorrhage, and irregular villus structure arrangement in the UC mice, which were more evident in the AD mice and were alleviated by BLF treatment (Figure 1C). These results showed that the UC model was successfully constructed, and the AD mice seemed more susceptible to colitis than the normal mice, while BLF effectively relieved colon tissue injury.

### 3.4 The IL‐1β, IL‐6, TNF‐α, and INF‐γ expression levels in mouse serum, brain, and colon tissues

**FIGURE 2:**
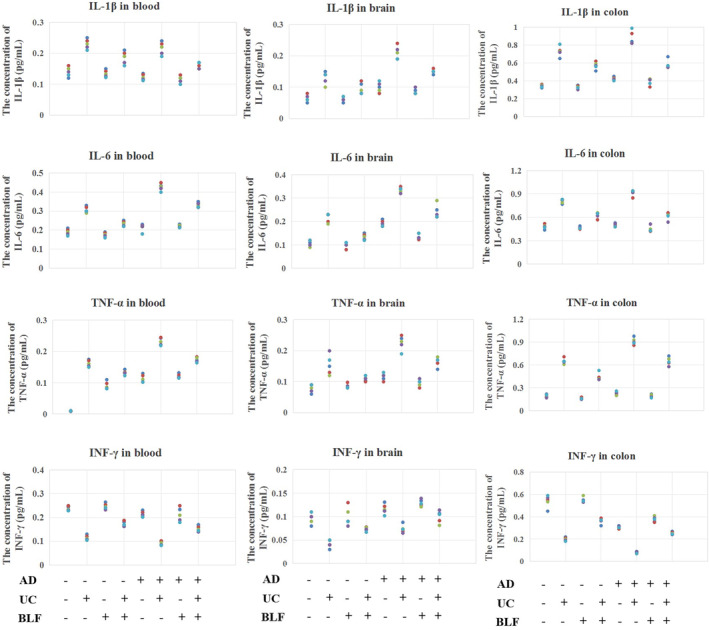
Detection of the inflammatory cytokines (IL‐1β, IL‐6, TNF‐α, and INF‐γ) in the mice serum, brain tissue, and colon tissue. *n* = 5.

To evaluate the impact of BLF on colitic damage, this study investigated the levels of certain cytokines in serum, brain, and colon tissues (*n* = 5). The results revealed significant IL‐1β, IL‐6, and TNF‐α pro‐inflammatory cytokine up‐regulation in the UC model group (*p <* 0.05, Figure 2), suggesting the presence of an inflammatory response in the mice with colitis. BLF administration significantly reduced the pro‐inflammatory factor expression levels (*p <* 0.05, Figure 2), which suggested that BLF treatment inhibited pro‐inflammatory cytokine expression, potentially attenuating the inflammatory response associated with colitis. Interestingly, the anti‐infective factor INF‐γ exhibited a contradictory tendency to the other three cytokines. The INF‐γ levels were more pronounced in the AD mice than the normal mice, indicating a dysregulated immune response in AD. This observation highlights the complexity of the immune system and suggests that AD may influence the immune response in colitis. Overall, BLF treatment demonstrated anti‐infective and anti‐inflammatory effects by suppressing the expression levels of pro‐inflammatory cytokines. Conversely, the dysregulation of the INF‐γ anti‐infective cytokine in the AD mice indicated the intricate interplay between AD and the immune response in colitis. The findings depicted in Figure 2 visually represented the changes observed in the levels of these cytokines after BLF treatment. In addition, the graph illustrates the significant reductions in the IL‐1β, IL‐6, and TNF‐α expression levels following BLF administration, further supporting the anti‐inflammatory effect of BLF in AD mice with colitis.

### 3.5 The MDA, GPX4, and 4‐HNE expression levels in the brain and colon tissues

**FIGURE 3:**
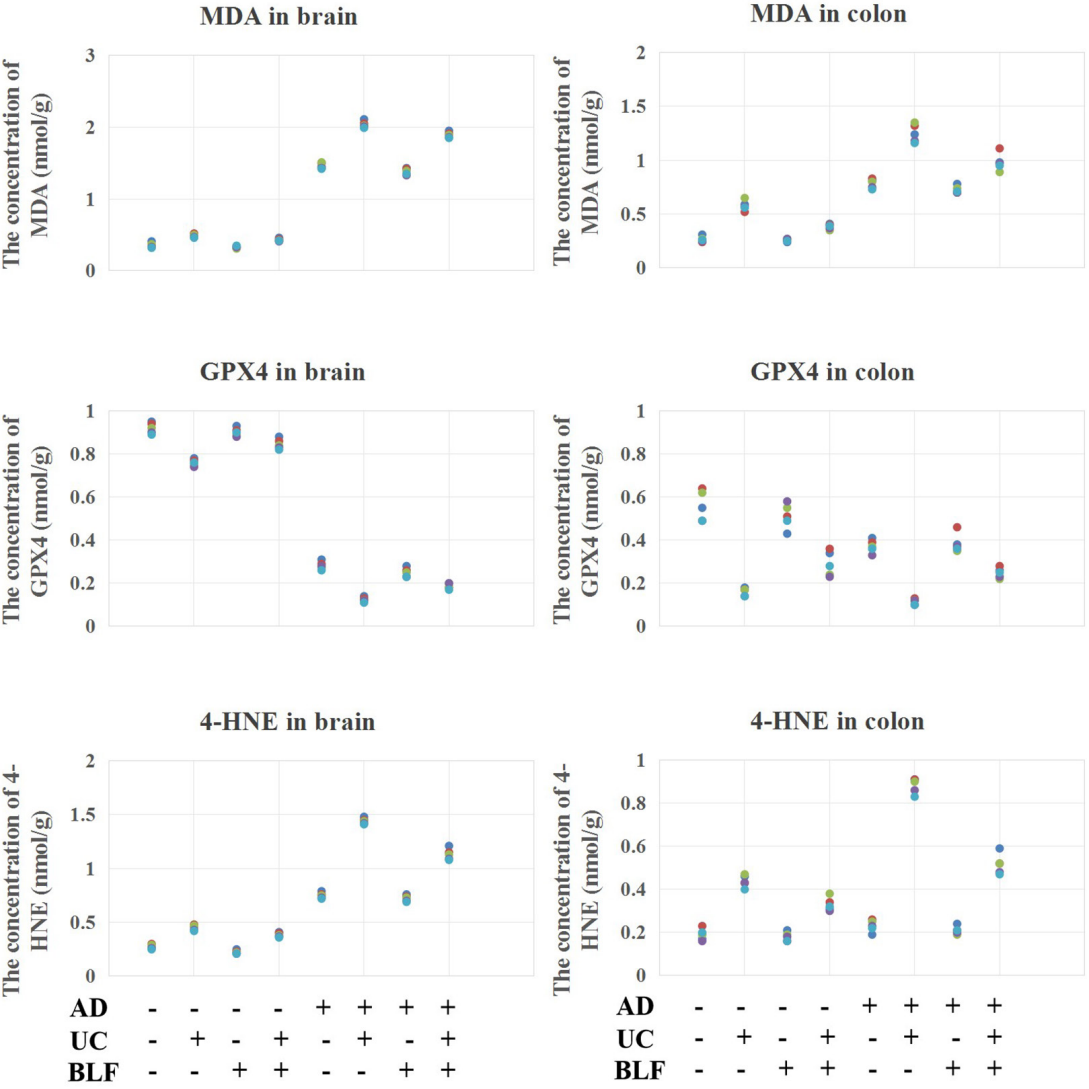
Detection of biochemical indicators (MDA, GPX4, and 4‐HNE) in the brain and colon tissue of the mice. *n* = 5.

**FIGURE 4:**
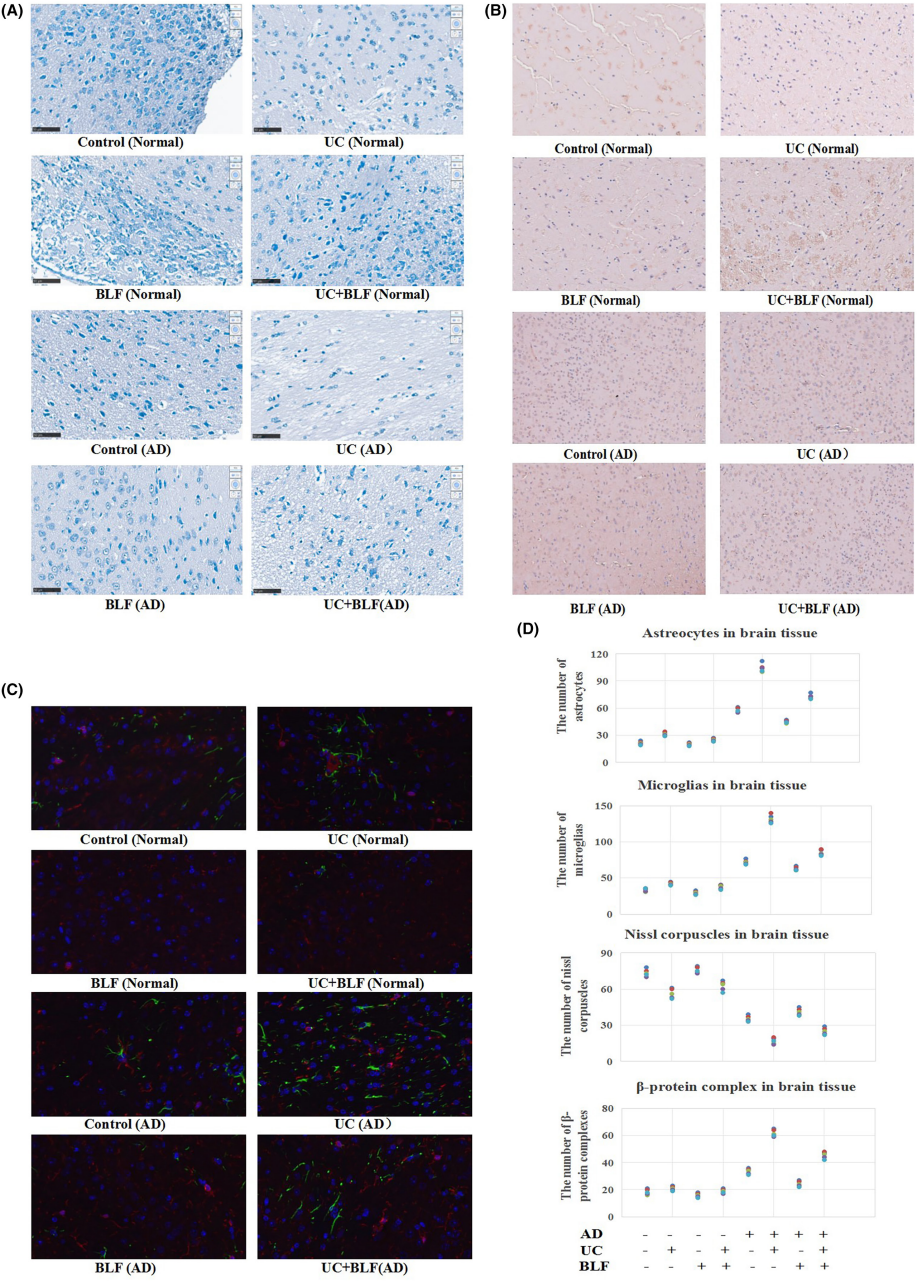
Special indicators staining in the brain tissue of the mice. (A) Nissl corpuscle staining of the mouse brains using toluidine blue. The images were captured at 200× magnification, *n* = 5. (B) The Aβ staining of the brain tissue via IHC. The images were captured at 200× magnification, *n* = 5. (C) The GFAP/Iba‐1 fluorescence double staining of the brain tissue. The images were captured at 400× magnification, *n* = 5. Blue (DAPI_350) denotes the cell nucleus, green (GFAP, FITC_488) signifies astrocytes, and red (Iba‐1, Cy3.5_594) represents microglias (MG). (D) Statistical analysis of the numbers of Nissl corpuscles, Aβ complexes, and astrocytes/microglias, *n* = 5. *p < 0.05 when compared to the control level. #*p <* 0.05 when compared to UC group.

This study assessed the lipid peroxide‐related index levels in both the normal and AD mice (*n* = 5). Compared with the healthy mice, the AD mice exhibited significantly elevated levels of these markers, suggesting that the disease escalated oxidative stress. Both tissue types in the AD mice displayed higher MDA and 4‐HNE levels than the control group, indicating oxidative damage upregulation. The elevation in MDA and 4‐HNE, widely recognized as lipid peroxidation markers, suggests increased oxidative damage in AD. Furthermore, the GPX4 level, an essential antioxidant enzyme, was significantly lower in the UC model group than in the control group (Figure 3), suggesting a compromised antioxidant defense mechanism in AD mice. The effect of BLF treatment on lipid peroxide‐related indices was investigated to explore potential interventions. The findings indicated a noticeable reduction in the levels of MDA and 4‐HNE following BLF administration. Although the levels remained higher than those observed in the control group, the decrease signified potential mitigation of lipid peroxidation via BLF treatment. Moreover, the GPX4 concentration was significantly upregulated in the BLF‐treated group compared with the UC group. This suggests that BLF may enhance the antioxidant capacity of the system since GPX4 plays a crucial role in detoxifying lipid peroxides and safeguarding cells against oxidative damage. Figure 4 visually represents the changes observed in the lipid peroxide‐related indices after BLF treatment. The graph demonstrates an apparent reduction in the MDA and 4‐HNE levels after BLF administration, indicating the potential of this treatment approach in alleviating the oxidative stress associated with AD development.

### 3.6 Nissl corpuscle staining of the brain tissue

As shown in Figure 4A, regardless of normal or AD mice, the total number of Nissl corpuscles in the UC model group was lower than in the control group, while BLF treatment increased this number to some degree. During injury or fatigue recovery, the nissite reappears, increases, and may reach normal levels. Therefore, Nissl corpuscles serve as a common marker of neuronal functional status. In this study, the decreased number of Nissl corpuscles in the UC model group further verified that colitis‐induced neuron damage and BLF alleviated both colon tissue and neuronal injury (Figure 4A,D).

### 3.7 Aβ staining of the brain tissue

The results indicated no significant differences between the Aβ expression of the control group and those treated solely with BLF. However, noteworthy findings were evident regarding the AD mice in the UC model, which exhibited Aβ up‐regulation. BLF administration reduced the Aβ expression levels of the AD mice, as shown in Figure 4B,D. This suggests that BLF may specifically affect Aβ protein expression regulation in AD. Furthermore, this indicates that BLF may impact the enzymes responsible for producing Aβ, decreasing the Aβ levels in mice with AD. The reduction in the Aβ expression after BLF treatment in AD mice provides insight into the potential use of BLF as a therapeutic agent for AD. The ability of BLF to reduce Aβ expression in AD mice indicates its potential to mitigate amyloid plaque accumulation and delay disease progression. In addition, by targeting Aβ production or aggregation, BLF can potentially interfere with the formation of these toxic plaques to preserve neuronal integrity and function. Moreover, the specific effect of BLF on Aβ expression in AD mice highlights its selectivity towards the pathological mechanisms associated with AD. This specificity suggests that BLF may possess a targeted mode of action, possibly interacting with the specific cellular pathways or signaling cascades involved in Aβ metabolism.

### 3.8 GFAP/Iba‐1 fluorescence double staining of the brain tissue

As shown in Figure 4C,D, both the GFAP and Iba‐1 signal intensities were upregulated in the AD mice with UC compared with the control. BLF reduced the GFAP and Iba‐1 protein expression levels, verifying that brain tissue inflammation and neuronal injury were evident in the UC model. BLF may cross the blood–brain barrier to inhibit inflammatory reactions and facilitate neuronal damage recovery (Figure 4C,D).

### 3.9 The effect of OT, homoorientin, VT, and IsVX on alleviating AD damage and UC injury

**FIGURE 5:**
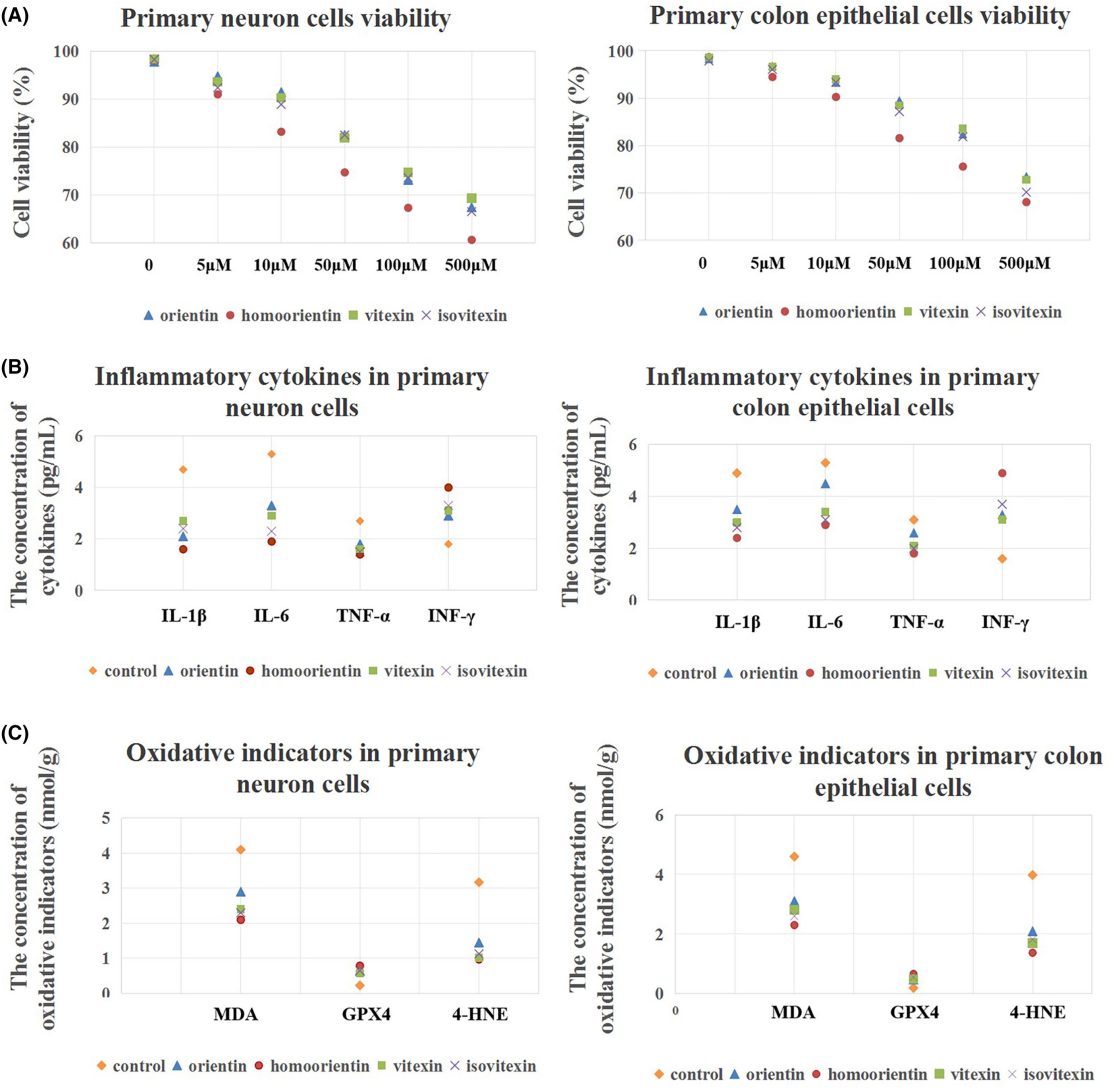
Detection of the cell viability, inflammatory cytokines, and biochemical indicators in the primary neuronal and colon epithelial cells. (A) The cell viability of the primary neuronal and colon epithelial cells was treated with the four BLF chemicals. *n* = 5. *p < 0.05 when compared to the untreated group (0 μm dosage group). (B) The inflammatory cytokine levels (IL‐1β, IL‐6, TNF‐α, and INF‐γ) in the primary neuronal and colon epithelial cells. *n* = 5. *p < 0.05 when compared to the control level. (C) The biochemical indicator concentrations (MDA, GPX4, and 4‐HNE) in the primary neuronal and colon epithelial cells. *n* = 5. *p < 0.05 when compared to the control level.

Results showed that homoorientin was more sensitive to these cells than the other three chemicals, requiring a 5 μm concentration for cell viability inhibition. Furthermore, 10 μm concentrations of the four chemicals significantly inhibited cell viability (Figure 5A). Considering the dosage selection principles of cell viability ≥80% (safety) and *p <* 0.05 when compared with the control (efficacy), this study selected 10 μm as the appropriate dosage of the four chemicals during the subsequent experiments. Detection of the inflammatory factors in the cells indicated that these four chemicals suppressed the IL‐1β, IL‐6, and TNF‐α expression levels and significantly increased that of INF‐γ, with homoorientin demonstrating the strongest bioactivity (Figure 5B). The four chemicals significantly downregulated the MDA and 4‐HNE oxidative indicator expression levels and upregulated that of the GPX4 antioxidative marker, which was consistent with the brain and colon tissue results. Homoorientin showed the strongest ability to regulate these indicators (Figure 5C).

### 3.10 The effects of embedded homoorientin on alleviating AD damage and UC injury

**FIGURE 6:**
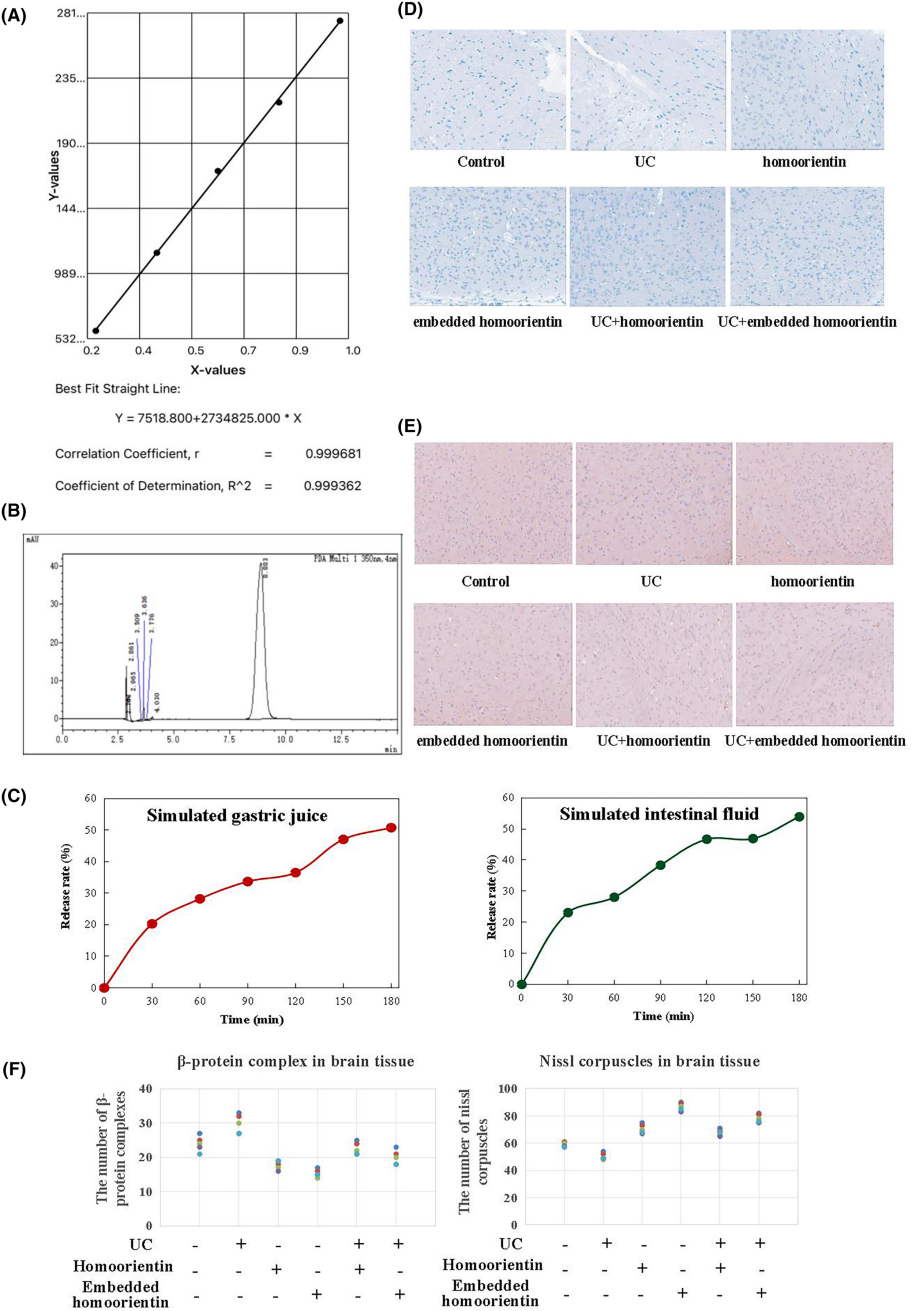
Detection of embedded homoorientin by HPLC and investigation of embedded homoorientin's effects in AD mice. (A) Standard curve of embedded homoorientin. (B) Detection of embedded homoorientin by HPLC. *n* = 5. (C) The *invitro* release simulation experiments of the nanoparticles in gastric and intestinal fluids, respectively. *n* = 5. (D) Nissl corpuscle staining of the mouse brains using toluidine blue. The images were captured at 200× magnification. *n* = 5. (E) The Aβ staining of the brain tissue via IHC. The images were captured at 200× magnification. *n* = 5. (F) Statistical analysis of the numbers of Nissl corpuscles and Aβ complexes in mouse brain tissue. *n* = 5. *p < 0.05 when compared to the control level. #*p <* 0.05 when compared to embedded homoorientin group.

As Figure 6A,B demonstrated, the retention time of homoorientin standard was 2.814 min. The compound was also detected in ursolic acid nanoparticles, indicating that ursolic acid successfully embedded homoorientin (Figure 6A,B). As shown in the Figure 6C, in simulated gastric juice, homoorientin were released rapidly in the first 30 min, with a release rate of 20.3%. They continued to be released for 60–150 min, and at 180 min, the release rate was 50.76%, gradually reaching its maximum value. The results showed that homoorientin embedded in nanoparticles had slow‐release properties in gastric juice, which was conducive to absorption. Homoorientin embedded in nanoparticles also exhibited slow‐release properties in simulated intestinal fluid, but their release rate was relatively fast compared with gastric fluid. At 120 min, the release rate reached 46.72%, followed by sustained release. At 180 min, the final release rate was 53.94%, and there was still a trend of sustained release (Figure 6C). As we expected, embedded homoorientin was verified to take stronger effects in alleviating AD damage when compared to homoorientin group, embodying on the decreased Aβ complexes and Nissl corpuscles (Figure 6D–F), which suggested that natural nanomaterial coating could enhance the bioavailability of homoorientin.

## 4 DISCUSSION

This study created chronic UC models utilizing two distinct strains of mice. One group consisted of typical, healthy mice, while the other comprised mice with AD. First, the UC injury of normal and AD mice was compared to determine whether AD mice were susceptible to colitis. Second, the effect of BLF on UC was investigated to determine whether BLF alleviated colitis injury. Third, the impact of BLF on AD was analyzed and compared with examine whether BLF has a beneficial effect on the two diseases via the regulation of several particular indices.

In addition, a preliminary study was conducted of the BLF molecular mechanism. Analysis of the changes in molecular sensors indicated that BLF might positively affect UC and AD via regulating various pathways. Firstly, BLF may alleviate UC damage by reducing the inflammatory response. The expression levels of the inflammatory cytokines decreased significantly after BLF treatment, which suggests that BLF may suppress inflammation and reduce colitis damage by modulating the immune response.^20^ Secondly, BLF may positively affect AD by inhibiting neuroinflammation and neuronal damage. This study showed decreased Aβ protein staining in AD mice after BLF treatment, which may be due to the inhibitory effect of BLF on neuronal inflammation and its protective impact on neurons. In addition, BLF may affect the function and structure of neurons by regulating the expression of specific biomarkers, such as Nissl bodies and GFAP/Iba‐1.^21^

Further research is required to explore the detailed BLF mechanism and determine its therapeutic potential for UC and AD. Furthermore, it is necessary to understand the interrelationship between UC and AD and their possible common pathological basis. Nevertheless, these findings will help understand the pathogenesis of UC and AD and provide a theoretical basis for developing new therapeutic strategies and drugs.^22^

Based on the average DAI index and organ index, a comparison between the UC damage in normal and AD mice indicated that the AD mice were more susceptible to DSS‐induced inflammation and injury. Furthermore, UC damage even accelerated AD development in mice, as shown by the Aβ protein staining. Regardless of normal or AD mice, BLF treatment significantly inhibited colitis injury and inflammatory reactions and suppressed AD progression to some degree, verifying that BLF presents a possible treatment for UC and AD. In the single UC model groups, specific biomarkers, including Nissl corpuscles and GFAP/iba‐1, expressed abnormally compared with the control group, further suggesting that patients with UC might be susceptible to AD. Thus, there might be common pathological bases, like cell signal pathways or molecular sensors, participating in the progression of the two diseases and regulating the related molecular mechanisms, which deserve our further research.

Hao et al. found that IL‐6 and TNF‐α levels decreased significantly in the huperzine A group and the low‐, medium‐, and high‐dose *Cornus officinalis* groups, while the IL‐10 level increased to varying degrees.^23^ Furthermore, this study confirmed that BLF regulated the expression levels of inflammatory factors. Intervention with isoforsythia glycoside was found to inhibit GFAP expression while promoting that of GPX4 in AD mice brains.^24^ These were consistent with BLF's bioactivity in present study, which also regarded GFAP and GPX4 as effective biomarkers of AD. Yang et al. used immunohistochemistry and Western blotting to determine Aβ expression level in brain tissue, the results indicated that GPX4 protein level increased, Nrf2/GPX4 aix was activated via salidroside intervention.^25^ Thus, the present study indicated that MDA, GPX4, and 4‐HNE might be sensitive indicators for AD progression.

In conclusion, this study verified the curative effects of BLF on two diseases, confirming that BLF suppresses AD risk in UC mice and UC deterioration in AD mice. Among four chemicals, homoorientin demonstrated the strongest effects in alleviating AD and UC, the embedded homoorientin coated with ursolic acid showed stronger bioactivities when compared to the uncoated group. Compared with the published articles, we found that there was very few studies of BLF complexes bioactivities in both AD and UC models, and for the first time, we validated that BLFs could inhibit the malignant progression of AD to UC. More interestingly, the embedded homoorientin was verified to take stronger effects than homoorientin in the present models, which was never reported in published articles. Therefore, as a novel natural therapeutic alternative, the efficacy of BLF complexes especially embedded homoorientin in clinical UC and AD patients deserve being revealed.

## AUTHOR CONTRIBUTIONS

Huiying Li conceived the study and supervised all the procedures. Huiying Li and Cuicui Jia wrote the raw text. Longyi Ran and Taiyu Zhang performed the animal experiments. Taiyu Zhang finished all the experiments in the revision stage. Jiarui Shi and Tsendsuren Amarmend performed the cell experiments. Cuicui Jia analyzed the data. Huiying Li and Taiiyu Zhang amended the manuscript. All data were generated in‐house, and no paper mill was used. All authors agree to be accountable for all aspects of work ensuring integrity and accuracy.

## FUNDING INFORMATION

This work was supported in part by “The Fundamental Research Funds for the Central Universities” (BLX202218).

## CONFLICT OF INTEREST STATEMENT

No known conflicts of interest associated with this publication.

## Supporting information


Tables S1–S2

## Data Availability

The data that support the findings of this study are available from the corresponding author upon reasonable request.
