# Supplementary material for: The alleviative effects comparison of four flavonoids from bamboo leaves on ulcerative colitis in an Alzheimer mouse model
Source: CNS Neurosci Ther. 2024 Feb 9;30(2):e14620. doi: 10.1111/cns.14620 (PMC10853884; doi:10.1111/cns.14620)
Supplement: Supplementary file 1 — Tables S1–S2 [file CNS-30-e14620-s001.pdf]

Supplementary Table 1 Disease activity indices (DAI)

Score	Weight loss (%)	Stool consistency	Occult or gross bleeding
0	(－)	Normal	Normal
1	1-5		
2	5-10	Loose	Guaiac (+)
3	10-15		
4	＞15	Diarrhea	Gross bleeding

1 Disease activity indices = sum of the weight loss, stool consistency, and bleeding scores/3.

2 Normal: well-formed pellets. Loose: pasty stools that do not adhere to the anus. Diarrhea: liquid stools that adhere to the anus. A Vernier caliper was used to

8.95±0.84[a]

determine the colon lengths.

Supplementary Table 2 The hematological parameters in the mouse blood of the different groups

Groups	WBC (10^9/ L)	Lymph (%)	Mid (%)	Gran (%)	RBC (10^{12}/ L)	PLT (10^9/ L)
Control (Normal)	3.11±0.23	69.27±6.83	7.22±0.35	7.17±0.50	6.41±0.63	583.07±55.71
UC (Normal)	5.08±0.61^{a}	89.27±11.40^{a}	9.42±0.78^{a}		6.06±0.45^{a}	511.33±65.58^{a}
BLF (Normal)	3.25±0.35^{b}	68.67±6.89^{b}	7.41±0.47^{b}	7.21±0.64^{b}	6.55±0.49^{b}	578.13±61.56^{b}
UC+BLF (Normal)	4.36±0.69^{ab}	76.58±7.39^{ab}	8.54±1.02^{ab}	8.13±0.97^{ab}	6.24±0.38^{ab}	567.45±57.18^{ab}
Control (AD)	3.26±0.45	74.17±8.26	7.50±0.72	7.64±0.49	6.72±0.65	569.21±56.84
UC (AD)	6.93±0.57^{a}	103.18±9.75^{a}	11.24±1.09^{a}	9.97±0.82^{a}	5.03±0.49^{a}	326.58±64.37^{a}
BLF (AD)	3.40±0.59^{b}	73.95±7.61^{b}	7.09±0.67^{b}	7.78±0.80^{b}	6.61±0.75^{b}	556.04±38.49^{b}
UC+BLF (AD)	5.53±0.67^{ab}	81.31±9.30^{ab}	9.15±1.07^{ab}	8.25±0.78^{ab}	6.16±0.56^{ab}	453.29±44.96^{ab}

The hematological data are represented as mean ± SD (n=5) and analyzed via one-way ANOVA. Based on a comparison between the same sex, a, $P<0.05$ vs. the Control group. b, $P<0.05$ vs. UC group.
